# Fertility intentions and the adoption of long-acting and permanent contraception (LAPM) among women: evidence from Western Kenya

**DOI:** 10.1186/s12905-019-0716-3

**Published:** 2019-02-01

**Authors:** Joshua Amo-Adjei, Michael Mutua, Carol Mukiira, Namuunda Mutombo, Sherine Athero, Alex Ezeh, Chimaraoke Izugbara

**Affiliations:** 1 University of Cape Coast, Cape Coast, Ghana; 2 African Population and Health Research Center, Nairobi, Kenya; 3 African Institute for Development Policy (AFIDEP), Nairobi, Kenya; 4 Population Services International, Lusaka, Zambia; 5 International Center for Research on Women, Washington, USA; 6 Community Health and Prevention, Drexel University, Philadelphia, USA

**Keywords:** Fertility intentions, Long-acting, Permanent contraception, Kenya

## Abstract

**Background:**

The use of long-acting and permanent method (LAPM) for family planning (FP) is of importance to the FP movement. A better understanding of how fertility-related intentions shape the usage of LAPM is important for programming. This paper explored the interaction of fertility intentions with LAPM use in rural western Kenya.

**Methods:**

We draw on monitoring data from 28,515 women aged 15–49 years who received FP services between 2013 and 2015 as part of a community-based FP project. We assessed the association between the use of LAPM and fertility intentions, adjusting for age, parity, education, service delivery model, FP counseling and year of data collection.

**Results:**

Of the 28,515 women who accessed FP services during the period (2013–2015), about two-thirds (57%) reported using LAPM, much higher than the national rates, and around 46% wanted another child within or after two years. In a multivariable regression model, women who desired no more children tended to use LAPM more than those wanting a child within or after some years as well as those uncertain about their future intentions.

**Conclusion:**

The significant rates of utilization of LAPM between both women who desired no more children and the fair proportion of use among women spacing births underscore the benefits of sustained community level interventions that address both the demand and supply barriers of contraceptive adoption and use.

## Introduction

As of 2015, half (50%) of all currently married women (15–49) in Kenya want to stop childbearing, yet only 28% of these women are using a modern contraceptive method [1]. Among those who want to stop childbearing, only 17% are using a long-acting contraception and 11%, a permanent method (LAPM) of contraception that would ideally suit their fertility intention by offering lifetime contraceptive protection. Low contraceptive uptake partly explains the proportion (35%) of unintended pregnancies in the country [1]. Unintended pregnancies can have serious implications for the health and wellbeing of mothers and children. High levels of unintended pregnancies can also frustrate the realization of development goals such as the Vision 2030 [2] and the Sustainable Development Goals (SDGs) [3]. The low uptake of contraceptives, particularly LAPM, among women who want no more children, highlights the need for addressing family planning (FP) issues, especially the need to advocate for appropriate methods that match their fertility intention. LAPM are also beneficial to women who want to have longer duration of birth spacing. It is particularly useful for young sexually active unmarried women who intend to delay children; it reduces repeat pregnancy in this population segment [4, 5].

In Western Kenya, the setting for this study, the gap between wanted fertility rate and achieved fertility rate is 2.0 compared to a difference of 1.2 at the national level [6]. Thus, women in this context have more children than they desire. Further, it is estimated that more than half (58%) of currently married women aged 15–49 years in this region, do not want to have any more children. Therefore, FP methods that best meet the fertility intentions of such women need to be availed to reduce the risk of unwanted pregnancies.

The causes of low use of modern contraceptives in Kenya and other similar settings include beliefs linking modern contraceptive use to promiscuity, fear of side effects, contraceptives as cause of infertility [7]. Poor quality of care from health care workers which discourages many women and their partners from accessing and using modern methods of contraceptives [8].

LAPMs are useful and important contraceptive options for allowing individuals and couples to achieve the desired number and spacing of children [9]. They are also safe, convenient, and cost-effective contraceptive methods, providing about 99% success rate [10, 11]. For women who experience distance as a barrier to service delivery points, LAPM reduces frequent visits to facilities for services. Although there is reasonably high level of knowledge about LAPM among currently married women [1], they are the least used method of contraception in several low and middle income countries (see, [12]), including Kenya. For instance, in the 2014 Kenya and Demographic and Health Survey (KDHS), 77% knew about IUD and 86% about implants but utilization of these methods remained significantly low [3.4% for IUD; and 26% for injectable] [1, 13].

Previous studies [14–16] have explored reasons for the uptake of LAPM. Besides the desire to have no more births due to the attainment of desired parity, other factors associated with the uptake of LAPM include perceived effectiveness of the methods [17, 18], the type of method (hormonal versus non-hormonal), fear or actual experience of side effects [19], and quality of family planning services provided [20]. This study further adds to the stock of evidence on this subject, particularly from the Kenyan context. To the best of our knowledge, there is dearth of evidence on fertility intentions and the use of LAPM in Kenya. This study contributes to filling this void.

## Materials and methods

### Study setting

Data were extracted from a family planning intervention conducted in two predominantly rural counties in Western Kenya, Busia and Siaya. Busia County is predominantly a Luhya community forms about 14.3% of the Kenyan population [21]. In Luhya custom, the extended family and clan units are very strong cultural institutions, demanding significant commitment from members. Although modernity has brought a lot of cultural shifts, men are still expected to pay in the form of cattle, sheep or goats or monetary equivalent for taking a wife. Intra-clan marriage still remains a taboo [22]. Siaya County on the other hand forms part of the larger Nyanza region with the Luo as the predominant ethnic group – about 12% of the Kenyan population [21]. While family formation begins late among men, it starts early for women; they are pro-natal and children born “belong” to the male partner in the Luo communities [23–25]. Patriarchy is a dominant aspect of social life in both communities, with men serving as ‘de facto*’* depositories of social and cultural power. These counties have an average total fertility rate of 5.5 despite a reasonably good level of modern contraceptive use overall (around 32% as at 2008 (the 2008 data is not disaggregated by county as was done in 2014 KDHS) and currently at 58% and 55% for Busia and Siaya counties) [1, 26]. In these areas, the injectable is the most commonly used contraceptive method (20% for Busia and 19% for Siaya respectively).

### Project description

Data for this study were extracted from a larger study on family planning intervention implemented in Busia and Siaya counties, Kenya. The full description of the project is detailed elsewhere (see, [27]). Briefly, from 2012 to 2014 the African Population and Health Research Center (APHRC) in partnership with the Ministry of Health, Kenya collaborated with five community organizations (Family Health Options-Kenya, Marie-Stopes, Kenya, Center for Study of Adolescence, Forum for African Women Educationalist, Christian Health Association of Kenya and Great Lakes University of Kisumu) to increase routine use of modern contraceptive methods among women of reproductive age. The intervention included deliberate efforts to influence women to switch from short-acting contraceptive methods (SACs) to long-acting reversible contraceptive methods (LAPM) or sterilization. Approximately 600 community health volunteers (CHVs) were recruited/engaged to distribute short-acting contraceptive methods, counseling of women, and referral of women for LAPM or sterilization services.

### Study design and data collection

This was a descriptive observational study – drawing data from routine service statistics. CHVs and service providers collected the data, using structured data collection tools as part of the monitoring processes at health facilities.

### Variables

The dependent variable was the contraceptive method the respondent was using during the reference year. This variable was recoded into a dichotomous variable: “1” if the respondent was using a LAPM (injectable, implant and IUD or opted for sterilization); and “0” if the respondent was on any other method other than LAPM. The primary independent variable was fertility intention, defined as whether or when a person wants to or not to have a child or children [28]. Studying fertility intentions enhances understanding of fertility behavior and improves reliability of fertility predictions [29]. In addition, we included several other variables known to be associated with contraceptive use such as: age, number of children, county (place of residence), and type of service delivery model (whether through mobile or stationary clinics), level of education, family planning counseling and project status. All these variables were reported by women at the point of care.

### Data analysis

The analyses focused on 28, 515 women aged 15–49 years who received FP and related services as part of the intervention between 2013 and 2015. At first visit, women were given a unique identifier so that regardless of the number of times they visited a facility, their primary records (e.g. age, parity etc.) remained unchanged. The exclusive identifiers helped to avoid double counting. Data analyses were conducted using STATA 13.0 (College Station, Texas 77,845 USA). Frequencies, cross-tabulations and logistic regression analyses were performed. Bivariate regression models were used to assess the crude association between each independent variable in influencing likelihood of using LAPM among service users. The main outcome variable was whether a woman received a LAPM on the current visit to a clinic or not. Exponential β (odds ratios) are used to depict the likelihood of receiving a LAPM compared to any other method of family planning. A ratio greater than 1 means greater likelihood of using LAPM than the reference category; a ratio less than 1 means lower likelihood of using LAPM than the reference category; and a ratio of 1 means same likelihood of using LAPM as the reference category. STATA version 13 was used to conduct the data analysis.

### Ethics

The project received ethics approval from the Kenya Medical Research Institute (KEMRI). Consent of individual women was waived given that the primary project was implemented in the form of service delivery rather than primary research.

## Results

### Characteristics of FP users

Table 1 shows the characteristics of 28,515 who received FP services from the project between 2013 and 2015. Half of the women (50%) were aged 25–39 years while 4 % were aged 40+ years. Twelve percent of the women had secondary or higher education, 41% had only some primary education, and 5 % had never attended school. Most (67%) of the women were from Siaya County and had not begun childbearing (53%). Over half (55%) of the women received FP from mobile health service providers. Under half (43%) of the women were new FP users through the project. Almost all (96%) the clients were counseled on FP before deciding on a method of contraception. More than half of the women reported using a long-acting method of contraception.

**Table 1:** Distribution of respondents by selected background characteristics

Background characteristics	%	Number of women clients
Fertility intention		
Wants no more children	6.6	1892
Wants another child within 2 years	9.1	2598
Wants another child after 2 years	37.0	10,547
Missing/Non-numeric response	47.3	13,478
Age		
15–24	45.9	13,078
25–39	50.3	14,331
40+	3.9	1106
Number of children		
0	53.0	15,125
1–2	17.7	5051
3–4	16.7	4770
5+	12.5	3569
County		
Siaya	67.3	19,197
Busia	32.7	9318
FP service delivery model		
Facility-based	44.9	12,792
Mobile service	55.1	15,723
Level of education		
None	4.7	1351
Primary incomplete	21.8	6220
Primary complete	18.9	5402
Secondary+	12.3	3512
Missing	42.2	12,030
Received counseling on family planning		
Yes	95.5	27,221
No	4.5	1294
Project status		
New FP user and project beneficiary	42.6	12,144
Existing FP user and new project beneficiary	21.8	6230
Existing FP user and project beneficiary	33.4	9529
Missing	2.1	612
Year of visit/service		
2013	15.0	4263
2014	62.5	17,823
2015	22.5	6429
Type of contraceptive method		
None	2.5	725
Short acting	10.8	3066
Injectable	29.7	8482
LAPM s	57.0	16,242
Total	100.0	28,515

### Descriptive bivariate association between background variables and use of LAPM

Table 2 shows the percent distribution of adoption of LAPM by background characteristics. In term of fertility intentions, most women who wanted no more children (85%) and wanted another child within two years (53%) and after two years (83%) often adopted LAPM. However, more than half of women who were uncertain – usually indicating “it’s up to God” or “can’t tell” tended to use injectable contraceptives (55%) more than any other method. Parity had differing association with the type of FP women chose – nulliparous (never had a child) women for instance utilized injectable (51%) more than LAPM (31%). On the contrary, almost all multiparous (5+ children) opted more for LAPM (90%). The FP service delivery model – facility-based or mobile showed differing pattern with whether LAPM or a short-acting method was adopted. For women who received services from facilities, injectable was prevalent (62%) while women served through mobile service usually chose a LAPM (79%). The majority (58%) of women who were counseled before a method preferred a long-acting method while the majority of the few (1294) who did not receive counseling chose injectable (65%).

**Table 2:** Distribution of respondents by contraceptive method adopted

Background characteristics	No method (725)	Short acting (*N* = 3066)	Injectable (8482)	LAPM (16,242)	N (28,515)	
Fertility intention						
Wants no more children	1.9	5.4	7.2	85.4		1892
Wants another child within 2 years	2.2	28.0	16.5	53.3		2598
Wants another child after 2 years	4.2	8.6	4.4	82.8		10,547
Missing/Non-numeric response	1.4	9.8	55.3	33.5		13,478
15–24	1.7	14.7	30.9	52.7		13,078
25–39	3.1	7.3	29.0	60.5		14,331
40+	4.6	9.1	25.4	60.8		1106
Number of children						
0	1.2	16.2	51.3	31.3		15,125
1–2	3.2	7.4	8.5	80.9		5051
3–4	4.0	3.9	4.1	87.9		4770
5+	5.2	1.7	2.8	90.3		3569
County						
Siaya	1.2	15.3	40.4	43.0		19,197
Busia	5.3	1.3	7.7	85.6		9318
FP service delivery model						
Facility-based	1.3	6.9	62.0	29.8		12,792
Mobile service	3.6	13.9	3.5	79.0		15,723
Level of education						
None	3.1	6.3	32.0	58.6		1351
Primary incomplete	4.0	2.7	6.3	87.0		6220
Primary complete	3.7	11.2	8.8	76.3		5402
Secondary+	2.5	34.7	7.2	55.6		3512
Missing	1.2	8.2	57.6	33.0		12,030
Received counseling on family planning						
Yes	2.6	11.1	28.1	58.2		27,221
No	1.0	3.3	65.1	30.5		1294
Project status						
New FP user and project beneficiary	0.3	17.3	23.3	59.1		12,144
Existing FP user and new project beneficiary	3.9	6.6	22.5	67.1		6230
Existing FP user and project beneficiary	4.6	5.6	40.4	49.4		9529
Missing	2.0	3.4	66.0	28.6		612
Year of visit/service						
2013	1.9	5.6	31.1	61.3	4263	
2014	2.4	8.3	31.0	58.3	17,823	
2015	3.4	20.9	25.4	50.4	6429	
Total	2.5	10.8	29.7	57.0		

### Factors associated with adoption of LAPM: Multivariable results

Table 3 contains three models used to clarify how fertility intentions affect the adoption of LAPM. Model 0 is the unadjusted estimates showing crude effects of each independent variable on the outcome variable. At least, one category of each of the independent factors showed significant association with the use of LAPM except fertility intentions. Despite that, the propensity to adopt a LAPM was higher among women who wanted their next child after two years in contrast to the reference category – wants no more children. In Model 1 where all the predictors, excluding fertility intentions were fitted into the equation, the signs for some variables turned negative. Particularly, increasing age and education reduced the likelihood of women using a LAPM (Table 3 Model 1). For instance, women who reported secondary or higher education were 0.21 (95% CI = 0.18–0.24) less likely to use a long-acting reversible method compared to their counterparts with no education. Also, the chances of using a LAPM among those 40+ years was around 34% lower than the 15–24 years group.

**Table 3:** Logistic regression odds of using LAPMs among women (15–49) in Siaya and Busia counties, Kenya

Explanatory variables	Model 0		Model 1		Model 2	
	OR	95% CI	OR	95% CI	OR	95% CI
Age (Ref: [15–24])						
25–39	1.53^***^	1.48, 1.59	0.96	0.87, 1.06	1.02	0.92, 1.13
40+	1.55^***^	1.38, 1.75	0.66^***^	0.53, 0.83	0.67^**^	0.53, 0.85
Number of children (Ref: None)						
1–2	4.24^***^	3.95, 4.55	5.22^***^	4.64, 5.87	5.06^***^	4.43, 5.79
3–4	7.30^***^	6.69, 7.96	7.02^***^	6.13, 8.03	6.16^***^	5.28, 7.19
5+	9.29^***^	8.31, 10.37	7.44^***^	6.29, 8.79	5.80^***^	4.81, 6.99
Level of education (Ref: None)						
Primary incomplete	6.71^***^	6.23, 7.22	0.51^***^	0.43, 0.59	0.64^***^	0.53, 0.77
Primary complete	3.22^***^	3.02, 3.43	0.36^***^	0.31, 0.42	0.45^***^	0.37, 0.54
Secondary+	1.25^***^	1.17, 1.34	0.21^***^	0.18, 0.24	0.25^***^	0.21, 0.31
County (Ref: Siaya)						
Busia	5.96^***^	5.62, 6.31	3.77^***^	3.36, 4.24	3.63^***^	3.22, 4.09
FP service delivery model (Ref: Facility-based)						
Mobile service	3.77^***^	3.63, 3.92	2.55^***^	2.29, 2.84	2.50^***^	2.23, 2.79
Project status (Ref: New FP user and project beneficiary)						
Existing FP user and new project beneficiary	2.04^***^	1.93, 2.15	0.72	0.64, 0.80	0.73^***^	0.65, 0.82
Existing FP user and project beneficiary	0.98	0.94, 1.02	0.38	0.34, 0.43	0.40^***^	0.35, 0.45
2014	1.40^***^	1.36, 1.44	1.43	1.26, 1.62	1.61^***^	1.41, 1.84
2015	1.02	0.97, 1.07	0.55	0.48, 0.64	0.65^***^	0.56, 0.76
Fertility intentions (Ref: Wants no more children)						
Wants another child within 2 years	1.14	1.06, 1.24			0.30^***^	0.24, 0.36
Wants another child after 2 years	4.80	4.57, 5.05			0.78^**^	0.66, 0.92
Missing/Non-numeric response	0.50	0.49, 0.52			0.62^***^	0.51, 0.77
Received counseling on (Ref: No)						
Yes	1.39	1.36, 1.43			1.15	0.91, 1.46

**p < 0.01,

***p < 0.001

The final model (2) presents the association between fertility intentions and adoption of LAPM net of the other independent factors. The model shows that having no intentions for more children has a strong significant influence on the use of LAPM. That is, wanting another child within or after two years considerably reduced the odds of using a LAPM by around 70 and 22% respectively. Similarly, women with uncertain intentions were 38% less likely to use a LAPM.

Model 2 further demonstrates that the project had higher influence on LAPM use. Compared to women who were newly introduced to FP use by the project, existing FP users and utilizing the project for FP were less inclined to use a LAPM. For the three years we collected data, utilization rates for LAPM were 61% higher in 2014 than in 2013.

## Discussion

This study used routine intervention monitoring data on 28, 515 women who adopted different FP methods. A key consideration of the project was to advance the use of LAPM to drive down higher than national-level fertility rates observed in the two counties. The study was motivated by a need to understand the drivers for LAPM, with interest on fertility intentions and to feed into or build on on-going interventions in order to sustain gains made in the project. Also, it provides evidence for similar projects to learn from, and design appropriate strategies to increase uptake of LAPM. Of the 28,515 participants who obtained services between 2013 and 2015, a majority (57%) adopted a LAPM, although a significantly smaller proportion (7%) wanted no more children. Our study reveals improving adoption of LAPM gauged against the low utilisation of LAPM at baseline [30].

The relatively high utilisation rates of LAPM among women, including those who had not completed children bearing – wanting more children within or after two years give an indication that the intervention had had beneficial influence on women in the zone of influence. Some previous studies from the African context (e.g. [31, 32]) have shown that the prevalence of LAPM use among women with incomplete fertility goals are quite low. Related studies from other parts of the world have shown similar results – less likelihood of LAPM use among women who wanted more children or were ambivalent about their fertility goals [6, 33]. This situation could be due to poor understanding of the types of LAPM availability and their effectiveness in helping women follow their fertility goals without interruptions. That is, if women are aware about the reversibility of most long-acting methods, their proclivity towards such methods will be positive regardless of their long or short-term goals. This was one of the goals of the project - to change negative narratives and misconceptions around LAPM as a method for only those who have completed child bearing or have achieved their desired fertility goals.

Following from the preceding, the findings highlight the peculiar importance of increasing LAPM through counselling prospective users. Evidence underscores the widespread misconceptions about LAPM in both developing [34, 35] and developed worlds [36, 37]. At the same time, evidence indicate that these misconceptions are linked to lack of awareness about the benefits and potential side-effects [37], which impede women and their couples use of LAPM. As our results reveal, women who received counselling were more inclined towards LAPM, re-echoing the need for service providers to offer comprehensive/adequate counselling to potential clients.

Our findings suggest that mobile FP service provision may be an important avenue to reach women with FP services. Mobile services have what it takes to increase easy access to FP commodities; they are more convenient and can be delivered at the doorstep of potential users. In resource-poor settings where access to general health services are limited, and also a function of several socioeconomic factors including, but not limited to income, mobile delivery of FP services may offer a great potential for increased utilization of FP methods overall, and LAPM too.

Some of our findings are also counterintuitive especially with respect to the association between level of education and LAPM utilization/adoption. In contrast to some previous studies [38], women with incomplete primary education were more likely to use LAPM than women with secondary and/or higher education. A possible reason for this finding may be that less educated women begin childbearing earlier [39, 40] and are therefore more likely to have attained their desired fertility than women with higher education. Consequently, less educated women may be more likely to use LAPM compared to highly educated women who will still be pursuing their fertility goals. On the other hand, the findings seem to affirm the emerging evidence in some African countries where educated women are increasingly turning to natural methods of family planning and in some instances, recurrent induced abortion to avert unplanned/unwanted births [41].

Our finding should be interpreted in the light of two major limitations: first, our study draws on service data, which most likely represent a highly self-selective group of women who may be quite different from the wider population of reproductive age women in the intervention zone. Nevertheless, women using any LAPM method are close to the proportion reported for the two counties in the 2014 KDHS, a nationally representative survey. This gives an indication of the minimal selection bias, which is often associated with service data. Similarly, by using facility-level data, we are unable to estimate statistical power of the sample used. However, given that the 2014 KDHS, which is a nationally representative survey, sampled 572 and 546 women from Siaya and Busia counties respectively [1], the number of women involved in this study help to offset some of the problems of statistical representativeness. This is in addition, likely to offset the challenges of missing data, mainly due to women’s unwillingness to disclose all information to service providers, or inability of these service providers to capture all requisite data.

## Conclusion

Our study revealed reasonably high levels of LAPM adoption among women, including a substantial proportion who had not completed child bearing or had intentions for additional births in the future. This is a pointer that future births may be planned barring method failure and method discontinuation. Within a context of an intervention to address demand and supply side barriers to LAPM adoption in developing communities, the findings give positive signs about the potential of addressing hurdles connected to LAPM adoption and utilisation.

## Abbreviations

CHVs: Community Health Volunteers
FP: Family Planning
KDHS: Kenya Demographic and Health Survey
LAPM: Long-Acting and Permanent Contraceptive Method
